# The effects of the recombinant YeaZ of *Vibrio harveyi* on the resuscitation and growth of soil bacteria in extreme soil environment

**DOI:** 10.7717/peerj.10342

**Published:** 2020-12-21

**Authors:** Yanlin Li, Jixiang Chen, Yonggang Wang, Dan Ma, Wenhong Rui

**Affiliations:** 1 School of Petrochemical Engineering, Lanzhou University of Technology, Lanzhou, China; 2 Chongqing Key Laboratory of Environmental Materials & Remediation Technologies/College of Chemistry and Environmental Engineering, Chongqing University of Arts and Sciences, Chongqing, China; 3 School of life science and engineering, Lanzhou University of Technology, Lanzhou, China

**Keywords:** Volcano land, Saline land, VBNC bacteria, YeaZ recombinant protein, Diversity

## Abstract

Numerous bacteria entered the viable but non-culturable state due to the stresses of dry and salt in soils. YeaZ of Gram-negative bacteria is a resuscitation promoting factor (Rpf) homologous protein could resuscitate bacteria of natural environment in VBNC state. To investigate the promoting effect of YeaZ on the isolation of viable but non-culturable (VBNC) bacteria from soil samples in extreme environments, the recombinant YeaZ of *Vibrio harveyi* was prepared and added to the soil samples from volcanic soil and saline soil in Northwest China. The study has shown that YeaZ can promote the recovery and growth of soil microorganisms, and the number of cultivable bacteria in volcanic and saline soil has increased from 0.17 × 10^3^ and 2.03 × 10^3^ cfu⋅ml^−1^ to 1.00 × 10^3^ and 5.55 × 10^3^ cfu⋅ml^−1^, respectively. The 16S rDNA gene sequencing and phylogenetic analysis showed that YeaZ played an essential role in the increase of composition and diversity of bacteria. A total of 13 bacterial strains were isolated from the volcanic soil samples, which belong to phyla Actinobacteria, Firmicutes and Gamma-proteobacteria. Four species, including *Ornithinimicrobium kibberense*, *Agrococcus citreus*, *Stenotrophomonas rhizophila* and *Pseudomonas zhaodongensis* were found in the control group, while *Micrococcus antarcticus*, *Kocuria rose*, *Salinibacterium xinjiangense*, *Planococcus antarcticus*, *Ornithinimicrobium kibberense* and *Pseudomonas zhaodongensis* were isolated from the treatment groups (addition of YeaZ). Twenty-one strains were isolated from the saline soil samples, including eight species from the control group and thirteen species from the treatment groups, among which nine species were only found, including *Bacillus oceanisediminis*, *Brevibacillus brevis*, *Paenibacillus xylanilyticus*, *Microbacterium maritypicum*, *B. subtilis*, *B. alcalophilus*, *B. niabensis*, *Oceanimonas doudoroffii* and *Zobellella taiwanensis*. The results suggest that addition of YeaZ to soil samples can promote the recovery of VBNC. This method has the implications for the discovery of VBNC bacteria that have potential environmental functions.

## Introduction

Many microorganisms inhabit the natural environments, but only 0.01–10% of these can be cultured using traditional techniques (Pace, 1997; Colwell et al., 1985; Oliver, 2010; Ding, Su & Yokota, 2011). In adverse environments, such as dry, hot and salt areas, it is difficult to isolate these bacteria by traditional techniques. Jookar et al. (2014) investigated the bacterial diversity of water, soil, sediment and salt samples from the east and western sites of Lake Urmia, Iran. The study is of great significance for exploring the extreme environmental microbial diversity, gene pool and the potential use of this information in biotechnology applications. Ma et al. (2009) studied the halophilic actinomycetes diversities of mud volcano and found that a large number of unknown microorganisms in Xinjiang, China. However, most of the bacteria in extreme environments cannot be obtained by using traditional pure cultivating method at present; most of them are in viable but non-culturable state and with low metabolic activities. But these metabolically active bacteria play an important role in soil nutrient cycling, biodegradation of pollutants, food safety, etc. (Su et al., 2013; Liu et al., 2016). These VBNC cells are intact and alive and can be resuscitated in favorable conditions.

A secreted protein, resuscitation promoting factor (Rpf) was first found in the supernatant of *Micrococcus luteus*, which has been considered essential for the resuscitation of dormant cells (Mukamolova et al., 1998). Rpf can resuscitate and promote the growth of Gram-positive and Gram-negative bacteria, including *Mycobacterium* spp., *Rhodococcus* spp., *Arthrobacter* spp., *Bacillus* spp., *Paenibacillus* spp. and *Curvibacter fontanus* (Ding & Yokota, 2010; Ding et al., 2012). It was also reported that the addition of the purified recombinant Rpf from *M. luteus* could recover the VBNC bacteria in the natural environment (Zou et al., 2014; Liu, 2007; Yu et al., 2015). Liu et al. (2016) reported that VBNC bacteria were isolated from sewage treatment systems using a culture supernatant from *M. luteus* containing Rpf. Puspita et al. (2015) reported the effect of *Tomitella biformata*-derived Rpf on the bacterial colony formation from a permafrost ice wedge, and found that the Rpf increased the number of colonies of Actinobacteria during the early stages of incubation, promoted growth of *T. biformata* from the permafrost ice wedge and exhibited cross species activity. In another study by Jin (2014), the total number of culturable bacteria increased from 7.5 × 10^6^ to 2.4 × 10^8^ cell/g of printing and dyeing wastewater samples by adding Rpf.. The number of culturable bacteria of pharmaceutical wastewater samples increased from 1.5 × 10^6^ to 2.4 × 10^8^ cell/g with in the presence of Rpf and the recovery rate of VBNC was (47.8–98.2)% (Zou et al., 2014).

Beside this, Rpf plays a significant role in enhancing the activity of bacterial communities in a polluted environment. Fida et al. (2017) found that a poly cyclic aromatic hydrocarbon (PAH) degrading bacterium *Novosphingobium* sp. LH128 of VBNC state were resuscitated by Rpf addition. A total of thirteen strains with heterotrophic nitrification ability were resuscitated by Rpf addition in a nitrogen polluted river (Su et al., 2019a). Su et al. (2019b) reported that Rpf from *Micrococcus luteus* may improve the degradative performance of bacterial populations and significantly enhanced phenol removal under high salinity stress. Ye et al. (2020) demonstrated that Rpf enhances PCB degradation by resuscitating PCB-degrading bacteria. Yu et al. (2020) revealed that the Rpf from *Micrococcus luteus* increased the abundances of Actinobacteria and Proteobacteria phyla which are involved in nutrient and phenol removal. Although there are many studies on the Rpf recovery of VBNC strains, the research on *yeaZ* recovery is relatively limited.

YeaZ is also a resuscitation factor like Rpf (Panutdaporn et al., 2006; Nichols et al., 2006), which has the similar stimulatory effect on bacterial resuscitation in several species, such as *Vibrio parahaemolyticus*, *Escherichia coli* and *Salmonella typhimurium* (Panutdaporn et al., 2006; Handford et al., 2009; Aydin et al., 2011). Previously, Li et al. (2016) cloned and expressed the *yeaZ* gene of *Vibrio harveyi* SF-1 and found that YeaZ has an indispensable effect on the resuscitation of VBNC. To investigate the promoting effect of YeaZ on the VBNC or uncultured bacteria in the extreme environment, two extreme environmental soil samples were selected, including volcanic soil and saline soil in Northwest China. The purified YeaZ of *V. harveyi* was prepared and added to the soil samples. The promoting effect was determined by using bacterial plate count and clone isolation methods.

## Materials and Methods

### Bacterial strains and culture media

*V. harveyi* SF-1 was isolated from diseased seabass (*Lateolabrax japonicus*) in China. The *yea* Z gene was cloned from the genomic DNA of *V. harveyi* SF-1, expressed in *E. coli* BL21 and purified by Ni^2+^-affinity chromatography, respectively (Li et al., 2016). The culture broth supplemented with pure activated or inactivated YeaZ protein was used for YeaZ treatment groups and the culture broth without YeaZ protein was used as the control group (Ding et al., 2012; Zou et al., 2014).

*E. coli* and other isolated bacteria were cultivated on Luria-Bertani (LB) nutrient agar containing 5.0 g peptone, 10 g yeast extract, 10 g sodium chloride (NaCl) and 20 g agar at pH 7.0 for 1,000 mL.

### Soil sampling

The study sites were located in the volcanic land (sample 1) of Xinjiang (89°30.779 E, 42°55.476 N) and saline land of Minqin (sample 2) of Gansu (103.64°E, 39.14°N) in China. Sample 1 is located in the northern edge of the Turpan Basin with a typical continental arid desert climate and the high air temperature (47.8 °C) in China. Sample 2 is located in the saline land of Minqin, which is located in the east of Tengger Desert, north of Inner Mongolia Badain Jaran Desert and west of the Qilian Mountains. The residues and impurities on the surface of the soil were cleaned up in the sample plots of volcanic soil and saline soil. A total of 6 soil samples (three random samples per environment) were taken from the surface soil (0–20 cm) by a 5-point sampling method. The samples were divided into 2 parts and put into sterile brown paper bags and brought back to the laboratory. One fresh soil sample was passed through a 2 mm sieve to determine the number of soil microbial functional bacteria, and the other was air dried and sieved to determine the physical and chemical properties of the soil. The physical and chemical properties of the soil are determined according to soil agrochemical analysis (Institute of Soil Science and Chinese Academy of Sciences, 1978). The soil moisture content is measured by the fresh soil drying method at 105 °C, and the soil pH value is measured by the glass electrode method with a water-soil ratio of 1:1 (Saul-Tcherkas, Unc & Steinberger, 2013). DNA was extracted from the soil samples with the genomic DNA extraction kit (Sangon Biotech Co. Ltd., Shanghai, China) after the soil samples were received in the laboratory.

### Bacterial plate count

The plate count method was used for determining the total number of culturable bacteria in the samples (Min et al., 2014). The LB liquid media supplemented with 10% activated or inactivated YeaZ protein (0.02 mg⋅mL^−1^) was used in treatment groups, and the LB liquid media without YeaZ protein was used as the control group. As previously described in Li et al. (2017), 10 g of each soil sample was suspended in 90 mL of sterile distilled water and was shaken for 20–30 min at 25 °C on a Thermostat oscillator. Soil sample solution (1%) was added to LB media with or without YeaZ protein, and was incubated at a constant temperature of 30 °C. After incubating for 12–24 h, the number of cultivable cells was detected by plate counting and spectrophotometry, and the dominant flora of different colony morphologies was selected for analysis.

### 16S rDNA gene sequencing and phylogenetic analysis

As previously described in Li et al. (2017), the total bacterial DNA was extracted with Ezup column bacterial genomic DNA extraction kit, and the 16S rRNA gene universal primers 27-F (5′-AGA GTT TGA TCC TGG CTC AG-3′) and 1492-R (5′-GGT TAC CTT GTT ACG CTT-3′) were used for PCR amplification. The PCR reaction system and the reaction cycle parameters refer to the description of Geetha & Manonmani (2010). The PCR products were detected by 1% agarose gel electrophoresis and sent to Shanghai Bioengineering Co., Ltd. for sequencing. The sequencing results were analyzed with the Basic Local Alignment Search Tool (BLAST) option of the National Centre for Biotechnology Information (NCBI) database (http://www.ncbi.nlm.nih.gov/). The sequences acquired in this study were deposited in the GenBank database (Accession no.: KY987120 –KY987153). Phylogenetic trees were constructed with MEGA 5.0 software using the neighbor-joining method (Tamura et al., 2011).

### Statistical analyses

The all experimental data was processed by Origin 8.0 software (OriginLab Corp., USA). One-way ANOVA and Duncan’s test were performed to assess the statistically significant differences of the number of total bacteria in control and treatment groups by using SPSS Base Ver.13.0 Statistical software (SPSS, IL, Chicago, the United States) (*P* < 0.05).

## Results

### Culturable bacterial counts

**Figure 1:**
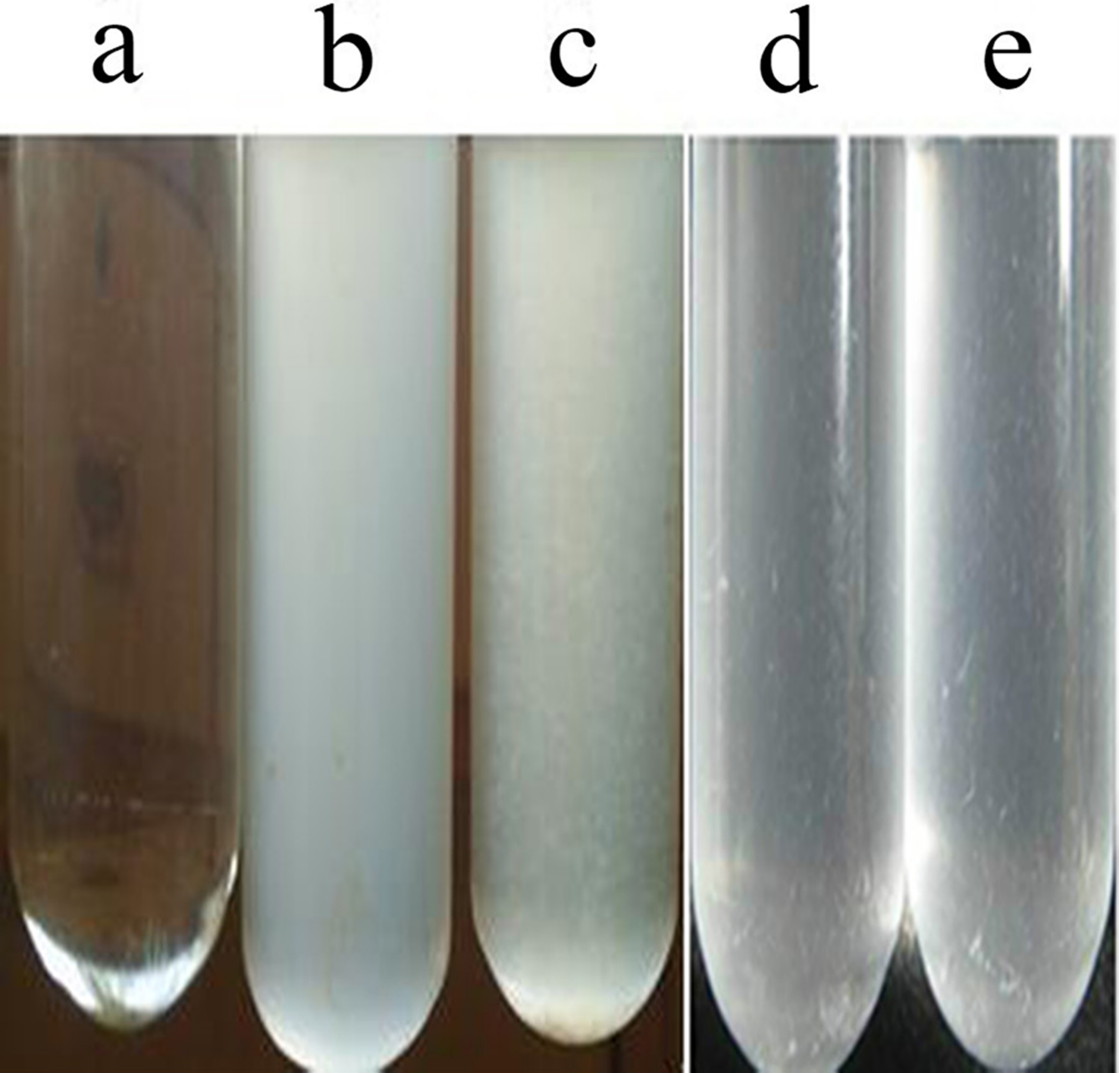
Growth of control and treatment groups. (A) Control; (B) saline land+activated YeaZ; (C) volcanic land+activated YeaZ; (D) saline land+inactivated YeaZ; (E) volcanic land+inactivated YeaZ.

After incubating for 13 h, the treatment groups became turbid (Fig. 1), indicated that YeaZ could promote bacterial recovery. The total bacterial populations of the volcanic land and saline land samples were shown in Table 1. The culturable bacterial counts in the volcanic land samples increased from 0.17 × 10^3^ cfu⋅ml^−1^ to 1.00 × 10^3^ cfu⋅ml^−1^ by addition of the activated YeaZ. Meanwhile, the culturable bacterial counts from the saline land samples increased from 2.03 × 10^3^ to 5.55 × 10^3^ cfu⋅ml^−1^ by the addition of activated YeaZ. This study defines the resurrection rates to represent the resuscitation and promoting results of recombinant YeaZ to the VBNC cells. The resurrection rates of the VBNC bacteria in volcanic and saline lands were 83.00% and 63.42%, respectively, and the recombination effect of YeaZ reached 5.88 and 2.73 times respectively in volcanic and saline lands, which showed the significant differences in bacterial populations between the treatment and the control groups (*p* < 0.05). The resuscitation of the VBNC bacteria with YeaZ of *V. harveyi* was displayed, and the number of culturable bacteria increased significantly after the addition of YeaZ in the soil samples.

**Table 1:** Number of total bacteria of control and treatment groups in agar plates. Resurrection rate (%) = (Total number of bacteria in treatment group-Total number of bacteria in control group) / (Total number of bacteria in treatment group × 100). Results are expressed as the mean ± standard deviation of three independent tests. One way analysis of variance (ANOVA) and multiple comparisons of significant differences were performed using Duncan’s test. Different letters in the same line meant significant difference at 0.05 level.

	Treatment group 1 (+YeaZ)	Treatment group 2 (+inactivated YeaZ)	Control group (-YeaZ)	Resurrection rate of VBNC bacteria (%)
Volcanic land	(1.00 ± 0.15) × 10^3^ a	(0.33 ± 0.15) × 10^3^ b	(0.17 ± 0.08) × 10^3^ b	83.00
Saline land	(5.55 ± 0.15) × 10^3^ a	(1.69 ± 0.10) × 10^3^ b	(2.03 ± 0.14) × 10^3^ c	63.42

**Notes.**

Resurrection rate (%) = (Total number of bacteria in treatment group-Total number of bacteria in control group) / Total number of bacteria in treatment group × 100.

Results are expressed as the mean ± standard deviation of three independent tests.

One way analysis of variance (ANOVA) and multiple comparisons of significant differences were performed using Duncan’s test. Different letters in the same line meant significant difference at 0.05 level.

### Diversities of the culturable bacteria

**Figure 2:**
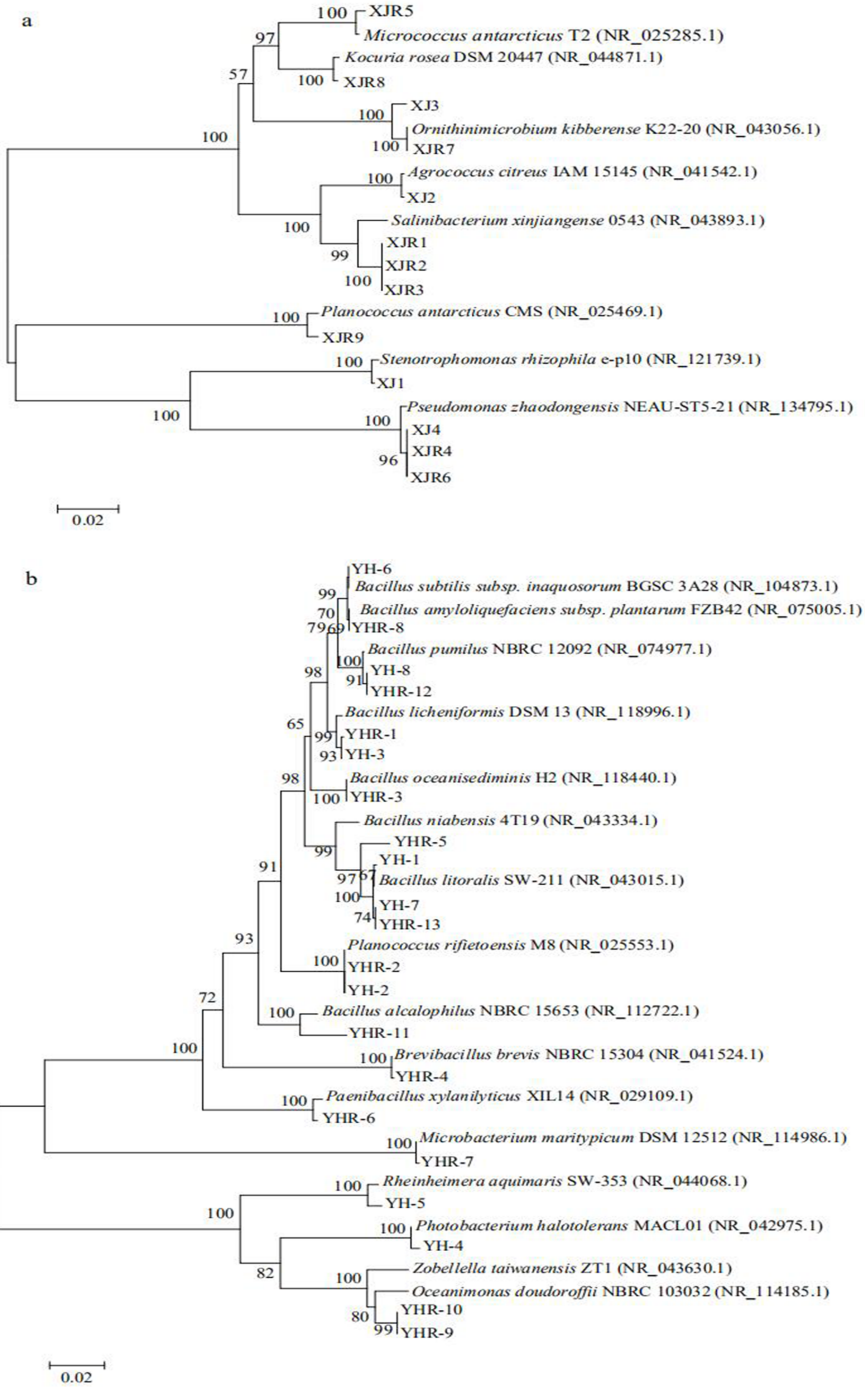
Phylogenetic trees of culturable bacterial communities of different soils. (A) Volcanic land; (B) saline land.

Thirteen bacterial strains were isolated from the soil samples of volcanic land, while twenty-one bacterial strains were obtained from the soil samples of saline land (Table 2). The bacteria from volcanic land soil belonged to three phylogenetic groups of Actinobacteria, Firmicutes and Gammaproteobacteria (Fig. 2A). Only four species, including *Ornithinimicrobium kibberense*, *Agrococcus citreus*, *Stenotrophomonas rhizophila* and *Pseudomonas zhaodongensis* were found in the control group, while further species were isolated from the treatment groups. *Micrococcus antarcticus*, *Kocuria rose*, *Salinibacterium xinjiangense* and *Planococcus antarcticus* were only found in the treatment groups.

**Table 2:** Genetic homology of 16S rDNA gene sequences of the isolated strains.

Volcanic Land				Saline Land			
Strain number	Closest type strain	Similarity (%)	Genbank accession number	Strain number	Closest type strain	Similarity (%)	Genbank accession number
XJR1	*Salinibacterium xinjiangense*	98	KY987120	YHR-1	*Bacillus licheniformis*	99	KY987133
XJR2	*Salinibacterium xinjiangense*	98	KY987121	YHR-2	*Planococcus rifietoensis*	100	KY987134
XJR3	*Salinibacterium xinjiangense*	98	KY987122	YHR-3	*Bacillus oceanisediminis*	100	KY987135
XJR4	*Pseudomonas zhaodongensis*	99	KY987123	YHR-4	*Brevibacillus brevis*	99	KY987136
XJR5	*Micrococcus antarcticus*	99	KY987124	YHR-5	*Bacillus litoralis*	98	KY987137
XJR6	*Pseudomonas zhaodongensis*	99	KY987125	YHR-6	*Paenibacillus xylanilyticus*	99	KY987138
XJR7	*Ornithinimicrobium kibberense*	99	KY987126	YHR-7	*Microbacterium maritypicum*	99	KY987139
XJR8	*Kocuria rosea*	99	KY987127	YHR-8	*Bacillus subtilis*	100	KY987140
XJR9	*Planococcus antarcticus*	99	KY987128	YHR-9	*Oceanimonas doudoroffii*	97	KY987141
XJ1	*Stenotrophomonas rhizophilo*	99	KY987129	YHR-10	*Zobellella taiwanensis*	97	KY987142
XJ2	*Agrococcus citreus*	99	KY987130	YHR-11	*Bacillus alcalophilus*	97	KY987143
XJ3	*Ornithinimicrobium kibberense*	99	KY987131	YHR-12	*Bacillus pumilus*	99	KY987144
XJ4	*Pseudomonas zhaodongensis*	99	KY987132	YHR-13	*Bacillus niabensis*	98	KY987145
				YH-1	*Bacillus litoralis*	99	KY987146
				YH-2	*Planococcus rifietoensis*	100	KY987147
				YH-3	*Bacillus licheniformis*	99	KY987148
				YH-4	*Photobacterium halotolerans*	99	KY987149
				YH-5	*Rheinheimera aquimaris*	98	KY987150
				YH-6	*Bacillus amyloliquefaciens*	99	KY987151
				YH-7	*Bacillus litoralis*	98	KY987152
				YH-8	*Bacillus pumilus*	99	KY987153

**Figure 3:**
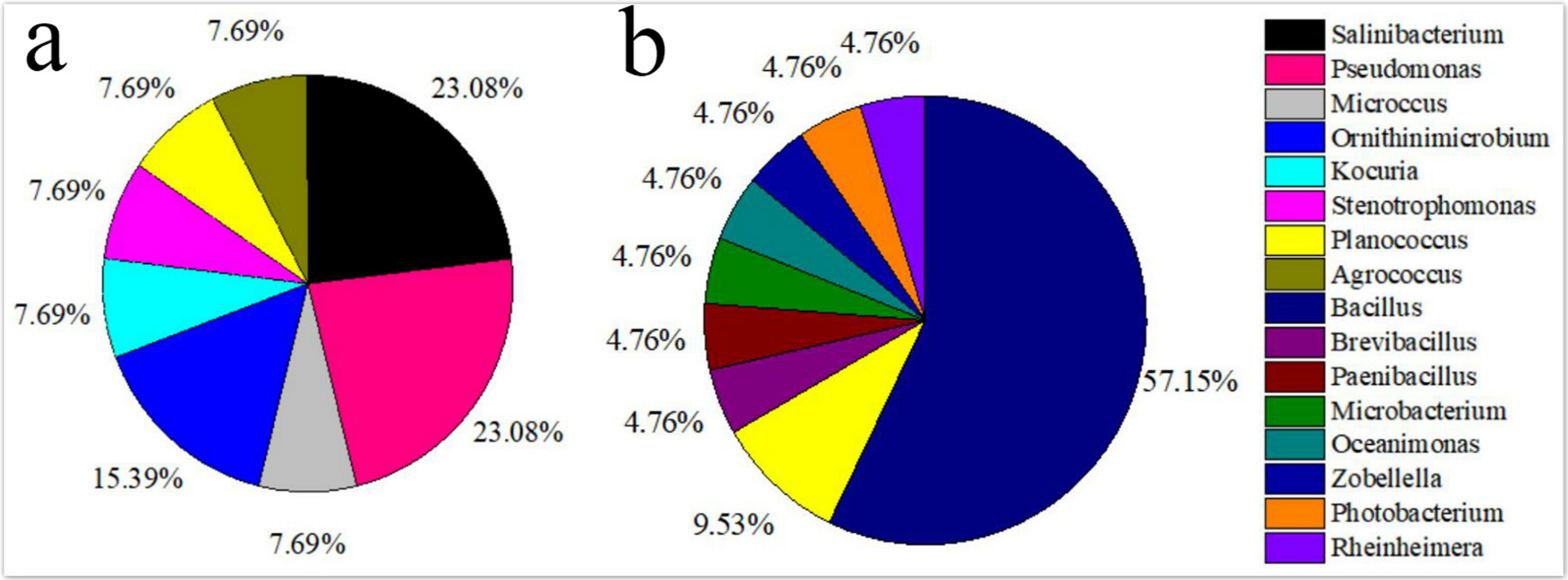
Relative genus abundance of the different soils. (A) Volcanic land; (B) saline land.

The isolated bacteria from the saline land soil also belonged to the phyla Firmicutes, Actinobacteria and Gammaproteobacteria (Fig. 2B). The majority of the bacterial species belonged to the genus *Bacillus*. Eight bacterial species in the control soil sample of saline land showed high similarities with *Bacillus litoralis*, *Planococcus rifietoensis*, *B. licheniformis*, *Photobacterium halotolerans*, *Rheinheimera aquimaris*, *B. amyloliquefaciens*, and *B. pumilus*. The culturable bacterial community in the treatment groups was more diverse than that of the control group. Nine species were only found in treatment groups, including *B. oceanisediminis*, *Brevibacillus brevis*, *Paenibacillus xylanilyticus*, *Microbacterium maritypicum*, *B. subtilis*, *B. alcalophilus*, *B. niabensis*, *Oceanimonas doudoroffii* and *Zobellella taiwanensis*. Among these, *Bacillus* species increased significantly. The abundance of each genus varied in the soil samples of volcanic land (Fig. 3A). *Salinibacterium*, *Pseudomonas* and *Ornithinmicrobium* were the predominant species and accounting for 23.08%, 23.08% and 15.39% in volcanic land, respectively. The distribution of different culturable bacteria from the saline land soil has shown in Fig. 3B. Bacillus was the most abundant group in the saline land, accounting for 57.15% of the total number.

## Discussion

The microorganisms of the extreme environments (volcanic and saline lands) tend to have a unique genetic type, which can produce special metabolites and bioactive substances. These microbes have a wide range of application values. The volcanic land of Xinjiang and saline land of Minqin are located in the special geographical location with special natural environmental conditions for the growth of high temperature, salt-tolerant and drought-resistant microorganisms. However, numerous bacteria could be in the VBNC state due to the extreme environmental stresses. The Rpf has been confirmed to be directly related to the resuscitation of VBNC bacteria. It not only has an effect on Gram-positive bacteria, but also has a good growth-promoting function for some Gram-negative bacteria. Yu et al. (2015) reported that the Rpf of Gram-positive *M. luteus* could increase the number of bacterial species in the soil of Xinjiang. The *rpf* genes of *M. luteus* are widespread among high G+C Gram-positive bacteria, such as *M. tuberculosis* (contains five *rpf* genes) (Downing et al., 2004). Rpf protein is similar to soluble lytic transglycosylases, which digests the peptidoglycan in the bacterial cell wall and the digested peptidoglycans may function as signaling molecules for the growth initiation and resuscitation of the VBNC bacterial cells (Mukamolova et al., 2006). The Rpf of *M. luteus* could mainly promote the growth of the Gram-positive bacteria. However, it also promotes the growth of some Gram-negative bacteria, such as *Curvibacter fontana* sp.

In previous study, Rpf-homologous protein, YeaZ had the stimulatory effect on bacterial resuscitation (Li et al., 2016). In this study, two types of soils from special natural environments were chosen to investigate the promoting effect of YeaZ from *V. harveyi* on the the presence of culturable bacteria. Sample 1 with the low moisture content (2.04%) and alkaline pH (8.50) from the volcanic land resulted in bacterial growth difficultly. On the other hand, sample 2 with the high moisture content (24.38%) and alkaline pH (9.65) from the saline land soil resulted in the abundance of bacteria. Because the bacterial quantity was most significantly related to moisture content (Saul-Tcherkas, Unc & Steinberger, 2013). The bacterial counts of culturable bacteria from soil samples in the Turpan Basin ranged from 0.29 × 10^3^ to 32.00 × 10^3^ cfu⋅g^−1^, and the culturable bacterial counts of volcanic land were 2.4 × 10^3^ cfu⋅g^−1^ (Pan, 2002). Crits-Christoph et al. (2013) reported that the number of culturable microorganisms in extreme aridity of the Atacama Desert varied from 3.1 × 10^3^ to 101 × 10^3^ cells⋅g^−1^ and the similar results were also observed in this study. As a resuscitation-promoting factor, YeaZ could resuscitate bacteria of the natural environment in the VBNC state. In this study, the culturable bacterial cell number increased significantly from 0.17 × 10^3^ to 1.00 ×10^3^ cells⋅g^−1^ in the volcanic land soils and also increased more than two times in saline soil by the addition of YeaZ (*p* < 0.05). Meanwhile, most of the isolated bacteria belonged to phyla Actinobacteria (61.54%) and Gamma-proteobacteria (30.77%) in the volcanic land. Similarly, previous studies also reported the prevalence of the phylum Actinobacteria in desert or cold desert ecosystem of McMurdo Dry Valleys, and Alpine and Arctic soil (Mccann et al., 2016; Horn et al., 2013; Rhodes et al., 2013). Zhang et al. (2015) reported that Gamma-proteobacteria was the most abundant group in the volcanic land of Xinjiang. Neilson et al. (2012) found that the communities were dominated by the phyla Actinobacteria and Chloroflexi in Atacama Desert, Chile. The predominant phyla were Firmicute (76.19%) and Gamma-proteobacteria (19.05%) in the saline soil and nine genera were only found in the samples by the addition of YeaZ. Abed et al. (2012) reported that Firmicutes were mostly detected in the saline lake sediments of Southern Australia. The soda lakes of the Kulunda Steppe, Russia with the highest salinity showed the poorest bacterial diversity, while Gamma-proteobacteria, Actinobacteria and Delta-proteobacteria were the dominant phyla in the lake sediments (Foti et al., 2008). The total bacterial populations were reported as (1.13–7.56) × 10^8^ cells⋅g^−1^ in the sediments of a hypersaline lake in Western Australia (Weigold et al., 2016). The results were similar with the researches above.

In the current study, the number and community diversity of culturable bacteria increased in the treatment groups, which suggested that the recombinant YeaZ could effectively promote the resuscitation of the Gram-positive and Gram-negative bacteria in the natural environments. Aydin et al. (2011) analyzed the YeaZ crystal structure of *Vibrio parahaemolyticus*, and the results showed that YeaZ protein is part of the HSP70 actin-like fold (HALF) protein family and is essential for the resuscitation of VBNC strains. The YeaZ proteins open up a new avenue for exploring new special flora and provide a scientific basis for addressing energy crisis and environmental governance by the resuscitation of VBNC bacteria.

## Conclusion

The results suggest that YeaZ can effectively promote the resuscitation and growth of the bacteria in the extreme environment. Recovering VBNC bacteria by YeaZ provides high opportunities to obtain more microorganisms with potential environmental functions.

##  Supplemental Information

10.7717/peerj.10342/supp-1 Data S1 Raw data for Table 1

## Additional Information and Declarations

### Data Availability

The following information was supplied regarding data availability: The data are available at NCBI: KY987120 –KY987153.
