# Supplementary material for: The effects of the recombinant YeaZ of Vibrio harveyi on the resuscitation and growth of soil bacteria in extreme soil environment
Source: PeerJ. 2020 Dec 21;8:e10342. doi: 10.7717/peerj.10342 (PMC7759134; doi:10.7717/peerj.10342)
Supplement: Data S1 [file peerj-08-10342-s001.doc]

|  | Treatment  group 1 (+YeaZ) | | | Treatment  group 2 (+inactivated YeaZ) | | | Control group (-YeaZ) | | |
| --- | --- | --- | --- | --- | --- | --- | --- | --- | --- |
| volcano land | 850 | 1140 | 1020 | 200 | 500 | 300 | 130 | 260 | 110 |
| saline land | 5690 | 5400 | 5560 | 1790 | 1680 | 1600 | 2170 | 2010 | 1900 |
